# Healthcare Providers’ Perception and Practice Toward Anaphylaxis in Children in the Qassim Region of Saudi Arabia

**DOI:** 10.7759/cureus.41366

**Published:** 2023-07-04

**Authors:** Yazeed A Alghasham, Kadi A Alhumaidi, Aeshah M Alharbi, Yasir S Alkhalifah

**Affiliations:** 1 Department of Pediatrics, Unaizah College of Medicine and Medical Sciences, Qassim University, Qassim, SAU; 2 College of Medicine, Unaizah College of Medicine and Medical Sciences, Qassim University, Unaizah, SAU; 3 Department of Pediatrics, College of Medicine, Majmaah University, Al Majma'ah, SAU; 4 Department of Pediatrics, Ministry of Education, Riyadh, SAU

**Keywords:** practical knowledge, medicine-pediatrics, allergy type 1, primary health care centers, primary health care providers, allergy and anaphylaxis

## Abstract

Introduction: Anaphylaxis is described as a severe allergic reaction, and prompt assistance is required by the patient to avoid any complications. The healthcare provider's knowledge plays an important role in the diagnosis and treatment of these cases. The aim of this study was to evaluate the knowledge among the primary healthcare providers working in the Qassim region of Saudi Arabia regarding the diagnosis and treatment of anaphylaxis.

Method: This cross-sectional study was conducted in the four different governments of the Qassim region of Saudi Arabia. The calculated sample size for the study was 119 primary healthcare providers. A self-administered questionnaire was devised to collect data regarding the diagnosis and treatment of anaphylaxis patients.

Results: Thirty-six (28.8%) out of 119 physicians were 25-30 years old, followed by 33 (26.4%) who were more than 40 years old; 53 (42.4%) and 47 (36.7%) had less than five and more than 10 years of experience, respectively. Age and experience were found to be significantly associated with knowledge about the diagnostic criterion (p=0.003 and p=0.000, respectively), while experience was significantly associated with the correct identification of signs and symptoms (p=0.031).

Conclusion: Knowledge about the diagnosis and management of anaphylaxis patients among primary healthcare providers was poor. Physicians are required to be educated to increase their level of knowledge to promptly diagnose and treat anaphylaxis cases.

## Introduction

Anaphylaxis is generally described as a severe allergic reaction with a rapid onset. It can affect healthy and young individuals and cause death within minutes [1]. According to the American Academy of Allergy, Asthma, and Immunology (AAAAI), anaphylaxis is an allergic reaction that can be life-threatening [2]. Therefore, every emergency care provider must have knowledge about it, to diagnose and treat these patients to minimize morbidity and mortality.

An increase in the incidence and prevalence of anaphylaxis over the last two decades has been reported in the literature [3]. Therefore, the National Institute of Allergy and Infectious Diseases and Food Allergy and the Food Allergy and Anaphylaxis Network (NIAID/FAAN) developed a diagnostic criterion [1]. In addition, clinical guidelines were also established to unify anaphylaxis patients’ management [4,5]. In 2014, a task force was formed to establish a practical recommendation for the management of anaphylaxis [6]. Despite all those guidelines and recommendations for the diagnosis and management of anaphylaxis patients, studies have reported the problems faced by healthcare providers in the correct identification and management of anaphylaxis [7,8]. 

Prognosis ignorance, either from the patient’s or doctor’s side, potentially causes negative outcomes [9]. Timely and correct management can prevent anaphylaxis-related deaths [10]. Studies conducted previously to evaluate healthcare providers’ knowledge and practice towards the management of anaphylaxis reported a lack of knowledge regarding the dose and route to administer the preferred drug as well as the selection of first-line medication to treat the emergency condition [11,12]. The findings of the studies revealed inadequate knowledge among healthcare providers regarding the management of anaphylaxis [2].

Besides the significance of having knowledge about the diagnosis and first-line treatment for the management of anaphylaxis patients, very limited studies have been conducted in Saudi Arabia to evaluate healthcare providers’ knowledge about the diagnosis and treatment of these patients. To the authors’ best knowledge, there were hardly any studies published from the Qassim region of Saudi Arabia to assess the primary healthcare providers’ knowledge and practice towards the management of anaphylaxis. Therefore, the present study was designed with the objective of assessing the knowledge of healthcare providers regarding anaphylaxis and evaluating the clinical practice among healthcare practitioners regarding the diagnosis and management of anaphylaxis.

## Materials and methods

This cross-sectional study was conducted from December 8, 2022, to March 15, 2023. The study was conducted in the Qassim region of Saudi Arabia, where primary healthcare providers participated. According to the Saudi statistical yearbook 2017, the Qassim region contains 181 primary healthcare centers (PHCs) [13]. However, the study was conducted in the four governments of the Qassim region out of 14 (Buraydah, Unaizah, AlRass, and Albukairyah). The selection of governments was according to the population and the number of primary health care centers.

In the four chosen governments, the total number of primary healthcare centers was 86. On average, each center usually has two general physicians. Therefore, the total number of physicians was 172, which served as a population for the sample size calculation. To calculate the sample size for the study, OpenEPi Version 3.01 was used. The total population size was 172, the chances of inclusion of any physician were 50%, and the confidence interval was set at 95%. Hence, by using the simple random sampling method, the calculated sample size for the study was 119.

To collect the required sample size, a convenient sampling method was used. In each of the selected governments, 50% of the primary healthcare centers (PHCs) were selected, and physicians working in those centers were invited to participate in the study. The inclusion criteria were that physicians of both genders could participate in the study. Any physician working outside the selected government was excluded from the study.

A self-administered questionnaire was devised for data collection by the authors of the study. The native language of the study authors was Arabic; therefore, the questionnaire was first prepared in Arabic and then translated into English. An English-language expert was requested for the translation. The Arabic questionnaire was then translated into English. The English version was translated back into Arabic to verify that the English version contained the actual meaning. After the translation, a pilot study was conducted to validate the questionnaire. A total of 15 responses were collected, and reliability analysis was performed. A high score from the analysis revealed that the questions included in the questionnaire were reliable, and no modification or exclusion of any question was required.

The questionnaire consisted of three sections. The first section was about demographics, which contained questions on gender, age, nationality, job description, and years of experience. The second part included questions regarding knowledge about anaphylaxis, such as signs and symptoms. The third part had questions on the physicians' clinical practice regarding diagnosing and managing anaphylaxis, which included knowledge about first-line medication, the recommended route, the preferred location for intramuscular injection, and the concentration and dose of epinephrine.

IBM Statistical Package for Social Sciences (SPSS) version 23 (IBM Corp., Armonk, NY, USA) was used for data analysis. A descriptive analysis of the data contained frequency distributions and bar charts. In inferential statistics, to study the relationship between two categorical variables, the Chi-square test or Fisher’s exact test is used. All p-values less than 0.05 were considered statistically significant.

Ethical approval was sought from the Regional Ethical Committee of Qassim Region, the Kingdom of Saudi Arabia, and participants were ensured confidentiality and the freedom to withdraw from the study at any time. Informed consent was obtained prior to filling out the questionnaire.

## Results

The total number of general physicians who participated in the study was 125. The proportion of male physicians (51.2%) was slightly higher compared to females (48.8%). Most of the participants were 25-30 years old (28.8%). The proportion of Saudi national doctors (57.6%) in the study was higher compared to non-Saudi nationals (42.4%). Participation from trainee doctors (54.4%) was highest, followed by specialists (32.8%). Regarding years of experience, most of them had less than five years of work experience (42.4%) (Table 1).

**Table 1:** Demographic characteristics of the study participants

Particulars	Frequency	Percentage
Age		
Less than 25 years	10	8
25 years - 30 years	36	28.8
31 years -35 years	22	17.6
36 years - 40 years	24	19.2
More than 40	33	26.4
Gender		
Male	64	51.2
Female	61	48.8
Nationality		
Saudi	72	57.6
Non-Saudi	53	42.4
Designation		
Medical student	7	5.6
Trainee	68	54.4
Specialist	41	32.8
Intern	9	7.2
Years of experience		
Less than five years	53	42.4
Five - 10 years	25	20
More than 10 years	47	37.6

**Figure 1:**
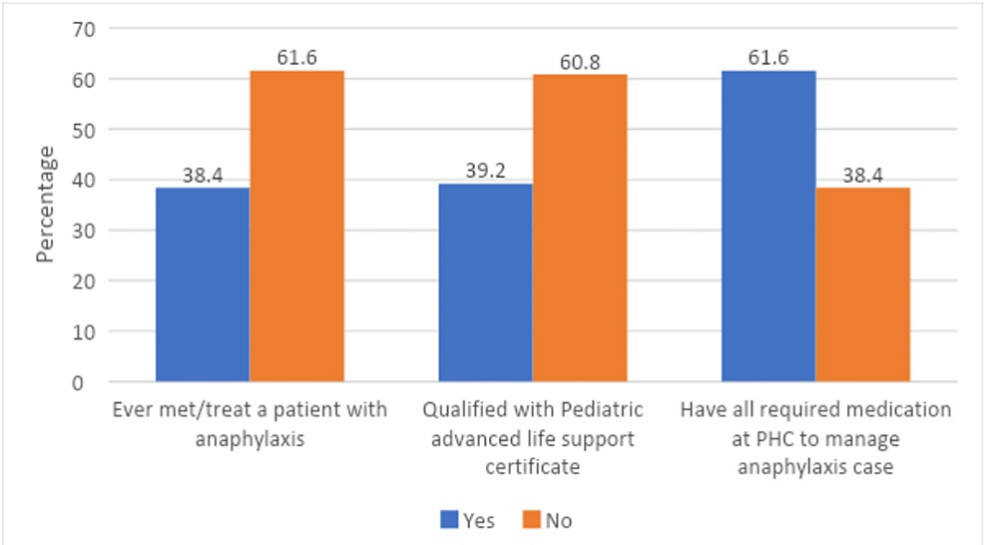
The proportion of positive and negative responses to the questions related to treating anaphylaxis patients, advanced life support certification, and the availability of anaphylaxis-related medication PHC: primary healthcare center

When physicians were asked about having any previous experience treating or meeting an anaphylactic patient, 61.6% responded negatively. In addition, over 60% of the doctors did not qualify for the advanced life support certification for pediatrics. Over 69% of the doctors spoke about the availability of mediation at the PHC to treat anaphylaxis cases (Figure 1).

Regarding the correct knowledge and practice for managing anaphylaxis patients, no question exceeded 70% of correct answers. In addition, when doctors were asked about the correct and preferred location (intramuscular) for the administration of epinephrine and the concentration and dose for epinephrine injection in pediatric patients, only 38.4% and 33.6%, respectively, gave the correct answer (Table 2).

**Table 2:** The proportion of correct answers related to knowledge and practice toward anaphylaxis

Knowledge	N(%)	
	Incorrect	Correct
Signs and symptoms of anaphylaxis	56 (44.8)	69 (55.2)
The type of hypersensitivity reaction anaphylaxis is	54 (43.2)	71 (56.8)
Practice		
The clinical criterion for diagnosing anaphylaxis	60 (48.0)	65 (52.0)
First-line medication in the treatment of anaphylaxis	50 (40.0)	75 (60.0)
The recommended route used to administer the preferred drug	42 (33.6)	83 (66.4)
The preferred location is an intramuscular injection of epinephrine	77 (61.6)	48 (38.4)
Intramuscular concentration & dose for epinephrine injection in pediatrics	83 (66.4)	42 (33.6)

**Figure 2:**
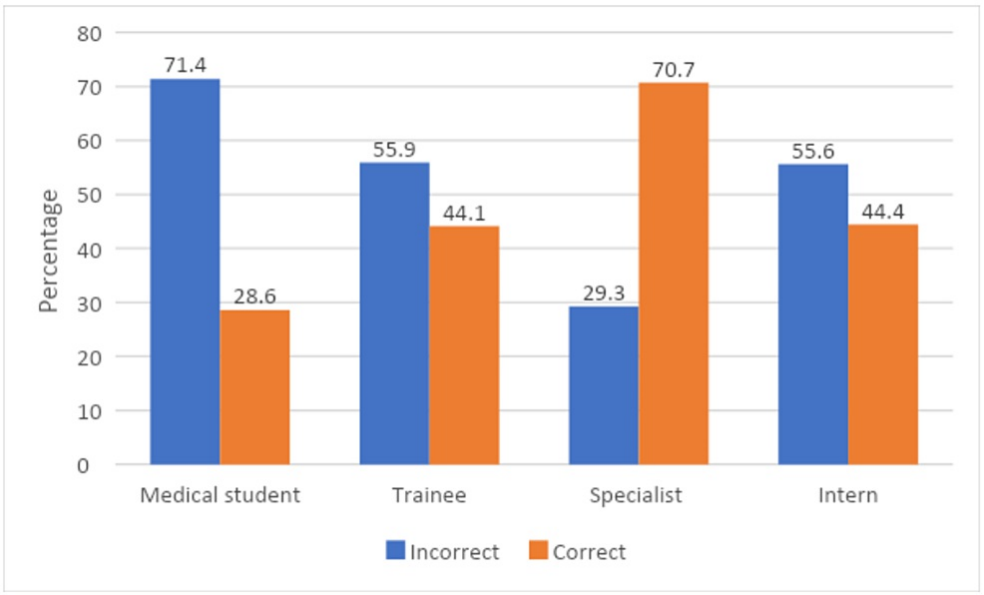
The association between the designation of the healthcare providers and the proportion of correct responses

Participants’ demographics were analyzed, along with their past experience, knowledge, and practice toward anaphylaxis. Over 70% of the specialists had correct knowledge about the clinical criteria for the diagnosis of anaphylaxis compared to trainees, interns, and students, which was significantly higher (p=0.024) (Figure 2).

When the age of the doctors was analyzed with the questions asked about knowledge and practice towards anaphylaxis, it was found that the experience regarding treating anaphylaxis patients was significantly higher among doctors more than 40 years of age (57.6%) (p=0.04). Similarly, regarding knowledge about the clinical criterion to diagnose anaphylaxis, doctors more than 40 years of age had a significantly higher proportion (72.7%) of correct knowledge compared to other age groups (p=0.003) (Table 3).

**Table 3:** The association between the age of the participants and their knowledge about anaphylaxis *Statistically significant at 0.05 level of significance

Ever met/treated a patient with anaphylaxis?	Age (in years), N(%)					p-value
	Less than 25	25-30	31-35	36-40	More than 40	
Yes	2 (20)	8( 22.2)	11 (50)	8 (33.3)	19 (57.6)	0.017^*^
No	8 (80)	28 (77.8)	11( 50)	16 (66.7)	14 (42.4)	
The clinical criterion for diagnosing anaphylaxis						
Incorrect	8 (80)	23 (63.9)	12 (54.5)	8 (33.3)	9 (27.3)	0.003^*^
Correct	2 (20)	13 (36.1)	10 (45.5)	16 (66.7)	24 (72.7)	

The work experience of the primary healthcare physicians who participated in the study was analyzed with questions related to anaphylaxis, and it was found that years of experience were significantly associated with previous experience treating anaphylaxis patients. Doctors with less than five years of experience had significantly fewer encounters with anaphylaxis patients (p=0.024). Similarly, these doctors were significantly fewer in number to have received advanced life support certification (p=0.039). In addition, knowledge about signs and symptoms and clinical criteria for diagnosis was found to be significantly associated with experience. Those who had more than 10 years of experience were significantly higher in number and had correct knowledge about signs and symptoms and clinical criteria for diagnosis (p= 0.031 and p=0.000), respectively) (Table 4).

**Table 4:** The association between the experience of the participants and their knowledge about anaphylaxis *Statistically significant at 0.05 level of significance

Ever met/treated a patient with anaphylaxis	Experience N(%)			p-value
	Less than five years	Five years - 10 years	More than 10 years	
Yes	13 (24.5)	12 (48)	23 (48.9)	0.024^*^
No	40 (75.5)	13 (52)	24 (51.1)	
Qualified with pediatric advanced life support certificate				
Yes	14 (26.4)	13 (52)	22 (46.8)	0.039^*^
No	39 (73.6)	12 (48)	25 (53.2)	
Signs and symptoms of anaphylaxis				
Incorrect	28 (52.8)	14 (56)	14 (29.8)	0.031^*^
Correct	25 (47.2)	11 (44)	33 (70.2)	
The clinical criterion for diagnosing anaphylaxis				
Incorrect	34 (64.2)	14 (56)	12 (25.5)	0.000^*^
Correct	19 (35.8)	11 (44)	35 (74.5)	

When study questions were analyzed with the nationality of the participants, it was found that 52.8% of non-Saudi and 27.8% of Saudi doctors had treated the anaphylaxis patients previously, and the proportion was significantly different (p=0.005). Similarly, having correct knowledge about the diagnostic criterion, 67.9% of non-Saudi doctors had correct knowledge compared to 40.3% of Saudi doctors, which was significantly higher (p=0.004) (Table 5).

**Table 5:** The association between the participants’ nationality and their knowledge about anaphylaxis *Statistically significant at 0.05 level of significance

Ever met/treated a patient with anaphylaxis	Nationality N(%)		p-value
	Saudi	Non-Saudi	
Yes	20 (27.8)	28 (52.8)	0.005^*^
No	52 (72.2)	25 (47.2)	
The clinical criterion for diagnosing anaphylaxis			
Incorrect	43 (59.7)	17 (32.1)	0.004^*^
Correct	29 (40.3)	36 (67.9)	

## Discussion

Anaphylaxis is a severe allergic reaction that needs to be diagnosed and treated promptly. Any delay in the correct diagnosis or providing appropriate treatment can cause death. Physicians must have knowledge about the diagnosis of anaphylaxis and its treatment protocols. Researchers have been conducting many studies to evaluate the level of knowledge among healthcare providers regarding the diagnosis and treatment of anaphylaxis. The present study was conducted with the same objectives: to evaluate the primary healthcare providers’ knowledge and practice regarding the management of anaphylaxis patients.

A high proportion of the study participants, which was over 60%, had neither treated nor met anaphylaxis patients before nor had advanced life support training. It was found that not all the participants responded correctly to the questions related to their knowledge of anaphylaxis. Around 70% of the study participants had correct knowledge about the signs, symptoms, and type of hypersensitivity reaction in anaphylaxis. Regarding patient management, the proportion of correct answers varied between 42% and 83%. In the study conducted by Drupad and Nagabushan to evaluate the knowledge about anaphylaxis among healthcare providers, it was found that around 65% of the participants had correct knowledge about the type of hypersensitivity in anaphylaxis [14]. Similarly, the study published by AlHaddad et al. reported a variation in correct answers between 30% and 100% [2].

Administering epinephrine is the next step after addressing the airway, breathing, and circulation [15]. In the present study, 75% of the physicians stated epinephrine as the first-line treatment for anaphylaxis. In the study conducted by Olabarri et al., epinephrine was chosen as the first-line treatment [16]. However, varying proportions have been reported to choose epinephrine as a first-line medication to treat anaphylaxis [17,18]. Parabhu and Yasmen reported that 90.1% of medical professionals prefer epinephrine as a first-line drug to treat anaphylaxis patients [19].

The correct and preferred route to administer epinephrine is intramuscular. From the findings of the present study, it was found that only 48% of the participants responded correctly to the questions. Other studies reported that a high proportion of physicians had correct knowledge about the preferred route to administer the drug [17,20]. Contrarily, Prabhu and Yasmeen reported that only 38.3% of the participants preferred the intramuscular route, while 51.7% preferred the intravascular route [19]. Similarly, in the study by Drupad and Nagabushan, the proportion of the participants who preferred to use the intramuscular route to administer the drug was as low as 16.5% [14]. 

Use of the correct dosage, which is 0.01 mg/kg, is another key, except for treating anaphylaxis patients. Perhaps the use of an incorrect dose prolongs the allergy and causes an unfavorable outcome. The present study's findings showed that only 42% of the primary healthcare physicians had the correct knowledge about the dosage. Similarly, a study from India reported that out of 265 tertiary care hospital doctors, only 40 had correct knowledge about the dose [14]. Another study conducted in two different district hospitals in England found a significant lack of knowledge among the physicians regarding the dose, route, and concentration of adrenaline [21]. Physicians are required to increase their knowledge about the management of anaphylaxis cases. Adherence to the guidelines regarding anaphylaxis patients’ management would improve knowledge as well as result in a successful outcome [7,22,23]. 

The healthcare providers working in hospital settings recognized advanced life support training as a significant factor in improving their clinical practice [24]. In addition to this, education should be provided to the patient’s family about the prevention and pre-hospital management of anaphylaxis, as well as instructions related to when and how to administer epinephrine [15]. Prompt administration of epinephrine is essential in the case of any new episode of anaphylaxis [25].

One of the study's limitations was that only cow-milk-related allergies were considered. Participants were not asked about the causes of anaphylaxis. Study participants did not evaluate their knowledge related to the initial steps taken for the suspected anaphylaxis case, post-treatment knowledge like recommended observation time, or educating patients regarding self-injectable epinephrine in case of reoccurrence. The small sample size was another limitation of the study. In addition, only primary healthcare doctors were included in the study; the inclusion of doctors from hospital emergencies could show different findings.

## Conclusions

The knowledge about the diagnosis and management of anaphylaxis among primary healthcare physicians working in the Qassim region was found to be very low. Because of the severity and shorter response time, physicians must be skilled to make a timely diagnosis and appropriately treat the patients. Therefore, it is highly recommended to educate physicians regarding the management of anaphylactic patients. It is also recommended to educate the family of the patient on how to administer the dose of epinephrine and avoid the risk factors that can cause any other episode of anaphylaxis.
